# 

**Published:** 1903-03

**Authors:** 


### PERSONALS.

The resignation of Dr. W. J. Muldown, Veterinarian 13th Cavalry, U. S. A., Fort Meade, South Dakota, has been accepted, to take effect January 14th.

Dr. P. A. Lehman, of York, Pa., was a JOURNAL office visitor in January. Dr. Lehman examines many cattle for the State Live-stock Sanitary Board that enter the southern part of Pennsylvania.

Dr. S. C. Freeland, of Marion, Kansas, is one of the proprietors of "The Rink Livery," and conducts an extensive proprietary remedy business in connection with his practice.

Dr. H. B. McDowell, of Middletown, Del., takes a keen interest in all State veterinary sanitary work.

Dr. H. S. Borneman, of Norristown, Pa., was a visitor to the JOURNAL office in February.

Dr. W. H. Dalrymple, of Baton Rouge, La., was a delegate to the Kansas City Convention of Live-Stock Growers and also in attendance at the several meetings held in Lincoln, Neb., and the representative of similar interests.

Dr. Albert Picquet, ex-army veterinarian, was a visitor to Philadelphia, Pa., in February.

Dr. V. B. Weaver., of Bangor, Pa., was a visitor to the JOURNAL office early in February and to the office of the State Veterinarian.

Dr. J. H. McKenzie, of Rochester, N. Y., who was so seriously injured by being kicked by a horse, causing a bad fracture of the leg, is slowly recovering.

Dr. James E. Lacey, of Titusville, Pa., practices his vocation minus one hand, having lost the same through the bite of one of his equine patients.

Dr. W. K. Lewis, of Chicago, Illinois, has returned to Anderson, S. C.,

Dr. Jas. W. Sallade, of Schuylkill Haven, Pa., was a visitor to Philadelphia in February.

Messrs. Hall & McCully is the name of a new firm now practising in 24th Street, New York City.

Dr. W. Horace Hoskins, of Philadelphia, Pa., addressed a joint session of the State Legislature of Delaware, early in February, relative to State veterinary control work, urging more stringent regulations and more extended work for the welfare of the "Blue Hen State."

Mrs. Dr. P. K. Jones, of Duquesne Heights, Pittsburg, Pa., died in the latter part of February from typhoid fever complicated with pneumonia.

Dr. W. H. Pendry, of Brooklyn, N. Y., has become a contributor to the columns of several newspapers, of experiences in his profession; they make interesting stories.

The Veterinary Department of the University of Pennsylvania entertained in February the Rt. Rev. Ethelbert Talbot, D.D., LL.B., at a reception in Houston Hall.

J. G. Ferneyhough, D.V.S., has been appointed State Veterinarian of Virginia.

Dr. E. B. Ackerman, of Brooklyn, N. Y., Chairman of the Committee on Intelligence and Education of the A. V. M. A., is doing very active work along the lines of the colleges and State Boards.

Dr. V. G. Hunt, of Arcola, Illinois, after more than forty years of active practice, is still active as a student of veterinary science, a reader and contributor to association work and journalism.

MULFORD'S BIOLOGICAL PRODUCTS, indicated in veterinary practice.

| | |
|---|---|
| ANTHRAX VACCINE.—Per tube of 10 complete doses, for cattle, horses and mules, or 20 complete doses for sheep and goats | $2 00 |
| (One complete dose consists of two injections of Vaccine, marked Nos. 1 and 2.) | |
| BLACK-LEG VACCINE.—Special Strength, No. 1 (sufficient to vaccinate 10 to 20 animals) | 1 25 |
| Special Strength, No. 2 (sufficient to vaccinate 20 to 40 animals) | 2 25 |
| Special Strength, No. 3 (sufficient to vaccinate 50 to 100 animals) | 5 00 |
| Special Strength, No. 4 (sufficient to vaccinate 100 to 200 animals) | 9 50 |
| Black-Leg Vaccinating Outfit | 5 00 |
| (Consisting of special veterinary hypodermic syringe, extra reinforced needles, tube for inserting vaccine, glass mortar, pestle and funnel, graduated measure, straining cloths; packed in neat polished hard-wood case.) | |
| ANTI-PNEUMOCOCCIC SERUM.—In vials of 20 c.c. | 2 75 |
| ANTI-STREPTOCOCCIC SERUM.—10 c.c. (immunizing dose) | 1 00 |
| 20 c.c. (curative dose) | 1 75 |
| IMMUNIZED SERUM FOR INFLUENZA AND DISTEMPER.—In vials of 20 c.c. | 1 00 |
| TETANUS ANTITOXIN.—For animals, 20 c.c. (curative dose) | 3 00 |
| Immunizing dose, 5 c.c. | 1 00 |
| N.B.—In ordering Tetanus Antitoxin, always specify "Veterinary" or "Human," as may be needed. | |
| MALLEIN.—In vials of 1 c.c., 1 injection | 35 |
| In vials of 2 c.c., 2 injections | 65 |
| In vials of 5 c.c. 5 injections | 1 50 |
| In vials of 10 c.c., 10 injections | 2 50 |
| TUBERCULIN.—In vials of 2 c.c., 2 injections | 35 |
| In vials of 5 c.c., 5 injections | 75 |
| In vials of 10 c.c., 10 injections | 1 25 |
| In vials of 25 c.c., 25 injections | 2 50 |
| In vials of 50 c.c., 50 injections | 4 50 |
| In vials of 100 c.c., 100 injections | 8 50 |
| In vials of 250 c.c., 250 injections | 20 00 |

Literature on Mulford's Biological Products, with full directions for use, supplied upon application.

H. K. MULFORD COMPANY, Chemists,

PHILADELPHIA. NEW YORK. CHICAGO.

